# Long-chain saturated fatty acids and its interaction with insulin resistance and the risk of nonalcoholic fatty liver disease in type 2 diabetes in Chinese

**DOI:** 10.3389/fendo.2022.1051807

**Published:** 2022-12-07

**Authors:** Li-Peng Jiang, Hong-Zhi Sun

**Affiliations:** ^1^ Department of Radiation Oncology, The First Affiliated Hospital of Jinzhou Medical University, Jinzhou, China; ^2^ Department of General Surgery, The First Affiliated Hospital of Jinzhou Medical University, Jinzhou, China; ^3^ Key Laboratory of Liaoning Tumor Clinical Metabolomics (KLLTCM), Jinzhou Medical University, Jinzhou, China

**Keywords:** myristic acid (14:0), palmitic acid (16:0), IR, NAFLD, T2D

## Abstract

**Introduction:**

This study aimed to explore relationships between long-chain saturated fatty acids (LSFAs) and nonalcoholic fatty liver disease (NAFLD) in patients with type 2 diabetes (T2D); and whether insulin action had an interactive effect with LSFAs on NAFLD progression.

**Methods:**

From April 2018 to April 2019, we extracted the electronic medical records of 481 patients with T2D who meet the inclusion and exclusion criteria from the Second Affiliated Hospital of Dalian Medical University. Ultrasound was used to estimate NAFLD at admission. Logistic regression analysis were used to estimate odds ratios (OR) and 95% confidence intervals (CI). The additive interaction was carried out to estimate interactions between LSFAs and insulin resistance (IR) in NAFLD patients with T2D.

**Results:**

Myristic acid (14:0) and palmitic acid (16:0) were positively associated with the risk of NAFLD (OR for myristic acid (14:0): 7.516, 3.557-15.882 and OR for palmitic acid (16:0): 4.071, 1.987-8.343, respectively). After adjustment for traditional risk factors, these associations were slightly attenuated but still highly significant. Co-presence of myristic acid (14:0)>72.83 μmol/L and IR>4.89 greatly increased OR of NAFLD to 9.691 (4.113-22.833). Similarly, co-presence of palmitic acid (16:0)>3745.43μmol/L and IR>4.89 greatly increased OR of NAFLD to 6.518(2.860-14.854). However, stearic acid (18:0) and risk of NAFLD have no association. Moreover, there was no association between very-long-chain SFAs (VLSFAs) and risk of NAFLD.

**Discussion:**

Myristic acid (14:0) and palmitic acid (16:0) were positively associated with the risk of NAFLD in T2D patients in China. High IR amplified the effect of high myristic acid (14:0) and high palmitic acid (16:0) on NAFLD.

## Introduction

NAFLD, a chronic liver disease, in recent years it has also been called Metabolic dysfunction-associated fatty liver disease (MAFLD). It is characterized by excess fat accumulation in the liver(more than 5%), unrelated to alcoholism (1), includes spectrum of liver diseases ranging from simple and benign steatosis to nonalcoholic steatohepatitis (NASH) (2, 3). NAFLD is becoming the commonest chronic liver disease (4, 5), 29.2% of the population is affected by NAFLD in china in 2019 (6). Not only in china, NAFLD is also a leading cause of liver disease globally (6, 7), in the U.S. alone, nearly 100 million people are affected by NAFLD (8), and this number is still growing, placing a heavy economic and public health burden on the economy and public health (4, 9). Up to one-third of patients with NAFLD are at risk of developing NASH (10), NASH has a high probability of progression to liver fibrosis and is a major risk factor for liver-related death (8).

Research has found that NAFLD was associated with the risk of type 2 diabetes (T2D) and its complications (11–13). South Korea conducted a five-year cohort study of non-obese, non-prediabetic male adults. Results showed that the NAFLD group had more subjects with impaired fasting glucose (IFG) and T2D than the non-NAFLD group (32.7% vs 17.6%; P < 0.05) (14). What’s more, a five-year cohort study in China confirmed that the adjusted RR (95%CI) for T2D was 4.462 (1.855-10.734, P < 0.001) in the NAFLD group compared with the non-NAFLD group, which means that NAFLD is T2D risk factors (15). NAFLD is better than BMI in forecasting the risk of T2D in Chinese subjects, and NAFLD may be an unrecognized risk factor in China’s recent increased incidence of T2D. The liver is an important organ for the metabolism of substances, energy and hormones, and can reduce or increase the blood sugar concentration together with muscle and adipose tissue (16). Although the liver has a huge compensatory ability, when liver steatosis leads to a certain degree of damage to liver cell function(such as NAFLD), the liver’s ability to regulate blood sugar will be reduced, and even insulin resistance and T2D will occur (17). NAFLD, or hepatic steatosis, is present in approximately 70% of T2D and almost all obese T2D (18–20), compared with obesity, the relationship of T2D as a risk factor for NAFLD is more direct and obvious (21). And even growing evidence suggested that there was a bidirectional association between NAFLD with T2D (22, 23). All of the evidence lending to “obesity, T2D and metabolic syndrome are consistently identified as the most important risk factors for NAFLD (6, 24)”. Therefore, the study of NAFLD in diabetic patients has potential biological and clinical significance.

In NAFLD, fatty acids accumulate in hepatocytes and reduce hepatic insulin sensitivity, thereby promoting hepatic gluconeogenesis, thereby increasing the risk of type 2 diabetes (T2D) or exacerbating disease pathology in diabetic patients (25, 26). There is growing evidence that links high in saturated fat with the increasing incidence of NAFLD (27). Saturated fatty acids (SFAs) are integrated biomarkers of diet and metabolism, and vary by SFAs chain length has unique metabolic and biological effects (28). Long-chain SFAs (LSFAs) are a unique group of SFAs with chain length ≥14, including myristic acid (14:0), palmitic acid (16:0) and stearic acid (18:0). Very-long-chain SFAs (VLSFAs) are a group of SFAs with chain length ≥20, including arachidic acid (20:0), behenic acid (22:0), and lignoceric acid (24:0). LSFAs and VLSFAs play vital roles in metabolic homeostasis. Research has shown that LSFAs and VLSFAs are associated with the risk of T2D (29–31). However, epidemiological studies of the association between LSFAs and VLSFAs and NAFLD were few. Fatty acid is associated with insulin action (32, 33) and those two have bidirectional impacts on prognosis (33). However, it is unclear whether the effects of fatty acids and insulin have an additive effect on the development of NAFLD under the context of T2D.

Therefore, using a cross-sectional study, we aimed to study 1) the associations between LSFAs and VLSFAs and the risk of NAFLD in T2D patients; 2) whether the effects of fatty acids and insulin have an additive effect on the development of NAFLD under the context of T2D.

## Methods

### Study population

A total of 1024 T2D patients were retrieved from April 2018 to April 2019, in the Second Affiliated Hospital of Dalian Medical University. Finally, according to the inclusion and exclusion criteria, 481 participants were included in the analysis and their electronic medical records were retrieved. The inclusion criteria included: 1) type 2 diabetes patients; 2) abdominal ultrasound scan; 3) plasma SFAs measurements. The exclusion criteria were: 1) below 18 years old; 2) secondary hepatic fat accumulation; 3) Alcoholic fatty liver disease(AFLD). T2D was diagnosed according to the 1999 World Health Organization’s criteria (34). This study was approved by the Clinical Research Ethics Committee of the First Affiliated Hospital of Jinzhou Medical University. Due to the retrospective nature of the study, informed consent was exempted, in line with the Declaration of Helsinki.

### Data collection and definitions

NAFLD was defined adopting the guidelines provided by the Chinese Association for Study of Liver Diseases (35). Briefly, fatty infiltration of the liver was evaluated by ultrasound examination. Participants without any other cause of liver disease and significant hepatic steatosis on the ultrasound were classified as non-NAFLD group. Waist circumference (WC), height and body weight were measured by specially trained nursing personnel using standardized methods. Subjects remain standing position after a gentle expiration. A tape measure was placed around the bare midriff of each subject and the WC measured midway between the lower border of the rib cage and the superior border of the iliac crest (36). Subjects were required to wear light clothes when measuring body weight and height. Body mass index (BMI) was calculated by dividing weight in kilograms by squared height in meters. According to the criteria recommended by the Working Group on Obesity in China, BMI was divided into four categories: underweight (<18.5 kg/m^2^), normal weight (18.5-23.9 kg/m^2^), obesity (24-27.9 kg/m^2^) and overweight (≥28 kg/m^2^) (37).We also extracted some other necessary clinical information including age, sex, duration of diabetes (years), alcohol intake, fasting insulin and fasting blood glucose. Insulin resistance (IR) was calculated as (fasting insulin*fasting blood glucose)/22.5 (38).

### Measurements of serum SFAs

Measurements of serum SFAs were conducted as described previously (39). Briefly, blood samples stored at -80°C were thawed at 4°C. Formic acid and ammonium acetate plus water or acetonitrile and isopropyl alcohol were used as mobile phase. C19:0 was used as internal standard. Eksigent LC100 and AB SCIEX Triple TOF 5600 (AB Sciex, Framingham, MA, USA) were used for LC-MS/MS analysis. Qualitative analysis was performed with PeakView1.2 (AB SCIEX). Quantitative analysis was performed with MultiQuant2.1(AB SCIEX).

### Statistical analysis

Continuous variables are expressed as means (standard deviations) when normally distributed or medians (interquartile ranges) when skewed; Categorical variables are presented as frequencies (percentage). Student’s *t*‐test (or Mann–Whitney *U*‐test when skewed) was used to compare differences in for continuous variables. χ^2^-test (or Fisher’s exact test where appropriate) was used to compare differences in categorical variables between participants with NAFLD and without NAFLD.

Binary logistic regressions were used to obtain odds ratios (OR) and 95% confidence intervals (CI) of SFAs for NAFLD in patients with T2D. Adjusting Models (multivariate model) were implemented to adjust for potential confounding factors for NAFLD. Confounding factors included age, gender, duration of diabetes, alcohol intake, BMI and WC. Sort the concentration of SFAs from smallest to largest and divide them into 5 parts with 20%, 40%, 60%, and 80% nodes. SFAs were analyzed as a five-categorical variable in three models, and p for trend was calculated to determine whether the OR value showed a corresponding linear trend with the change of SFAs concentration.

Additive interaction was used to estimate interactions between LSFAs and IR (40). Three measures including relative excess risk due to interaction (RERI), attributable proportion due to interaction (AP) and synergy index (S) were implemented to estimate additive interactions. A significant RERI>0, AP>0 or S>1 suggested a synergistic effect or additive interaction between LSFAs and IR for NAFLD.

Sensitivity analysis was conducted to check the consistency of the results after exclusion of subjected with a history of alcohol use. The SAS (9.4) was used to carry out the data (SAS Institute Inc., Cary, North Carolina, USA) unless otherwise specified. It is statistically significant that a two-tailed *p* value <0.05.

## Results

### Characteristics of the study participants

In the cross-sectional study, we collected 418 confirmed participants of T2D with or without NAFLD (280 cases with NAFLD and 138 cases without NAFLD). Baseline characteristics of NAFLD and non-NAFLD subjects are shown in **Table 1**. The 418 subjects had a mean age of 58.6 (SD:13.0) years, mean BMI of 26.7 (SD: 3.8) kg/m² and mean WC of 93.4 (SD: 9.4) cm. The participants with NAFLD had a younger age, larger WC and heavier weight and longer duration of diabetes by comparing with their counterparts non-NAFLD. In addition, alcohol intake was significantly different between NAFLD groups and non-NAFLD groups, however the p-value of the statistic is close to 0.05 and needs careful consideration. LSFAs including myristic acid (14:0), palmitic acid (16:0) were also significantly different between the NAFLD group and the non-NAFLD group. The p-value of stearic acid (18:0) is close to 0.05 and needs careful consideration.

**Table 1:** Baseline characteristics of participants with or without nonalcoholic fatty liver disease.

Variables	Non-NAFLD (138)	NAFLD (280)	*P*-value
Age (years)	60.66+12.57	57.53+13.13	0.021
Male	76 (55.07)	122 (43.57)	0.027
WC (cm)	90.12+9.29	95.03+9.05	0.000
Weight (kg)	69.00 (60.00,77.00)	74.00 (66.00,85.00)	0.000
Height (cm)	165.00 (160.00,173.00)	165.00 (160.00,173.00)	0.976
BMI (kg/m^2^)	24.79 (22.58,26.87)	26.94 (24.67,29.74)	0.000
BMI<18.5	1 (0.72)	0 (0.00)	
BMI≥18.5 and <24	49 (35.51)	52 (18.57)	
BMI≥24 and <28	62 (44.93)	112 (40.00)	
BMI≥28	26 (18.84)	116 (41.43)	
Duration of diabetes (years)	7.50 (2.00,15.00)	11.00 (5.00,18.00)	0.001
Alcohol intake			0.048
Ex-drinker	9 (6.52)	6 (2.14)	
Current drinker	12 (8.70)	18 (6.43)	
Non-drinker	117 (84.78)	256 (91.43)	
IR	3.93 (2.10,6.93)	5.42 (3.51,9.24)	0.000
LSFAS (μmol/L)			
14:0 (myristic acid)	44.01 (25.31,63.55)	73.10 (44.10,118.97)	0.000
16:0 (palmitic acid)	3038.06 (2601.15,3719.74)	3680.69 (2941.36,4730.22)	0.000
18:0 (stearic acid)	941.78 (812.76,1206.18)	1027.83 (838.43,1263.82)	0.047
VLSFAS (μmol/L)			
20:0 (arachidic acid)	5.65 (3.72,8.30)	5.90 (4.02,7.96)	0.418
22:0 (behenic acid)	1.47 (0.80,2.71)	1.56 (0.91,2.85)	0.322
24:0 (lignoceric acid)	1.51 (0.99,2.58)	1.42 (0.98,2.47)	0.988

Data are mean (standard deviation), median (interquartile range) or n (%). WC ,Waist circumference; BMI, body mass index; IR, insulin resistance; LSFAs, long-chain saturated fatty acids; VLSFAs, Very-long-chain saturated fatty acids; NAFLD, nonalcoholic fatty liver disease.

Overall, the concentrations of LSFAs in the NAFLD group were higher than their counterparts in the non-NAFLD group. However, there was no significant difference between the NAFLD group and the non-NAFLD group for VLSFAs (arachidic acid (20:0), behenic acid (22:0), lignoceric acid (24:0)).

### Associations of SFAs with NAFLD

The ORs for NAFLD by quintile of plasma SFAs relative to the lowest quintile are shown in **Table 2**. Myristic acid (14:0) and palmitic acid (16:0) were positively associated with the risk of NAFLD in univariate analyses. After adjustment for age, gender, duration of diabetes, alcohol intake, the positive associations were still significant. After further adjustment for BMI, WC, the effect sizes of myristic acid (14:0) and palmitic acid (16:0) remained (OR for myristic acid (14:0): 7.072, 3.191-15.673 and OR for palmitic acid (16:0): 3.889, 1.783-8.481, respectively). However, stearic acid (18:0) and risk of NAFLD have no association. Interestingly, there was no association between VLSFAs and risk of NAFLD.

**Table 2:** Odds ratios (and 95% CIs) for nonalcoholic fatty liver disease by quintile of saturated fatty acids.

Variables	Quintile 2	Quintile 3	Quintile 4	Quintile 5	*p* for trend	Per 1 SD
LSFAs						
14:0 (myristic acid)						
Univariable Model	1.694 (0.920,3.118)	2.495 (1.333,4.669)	5.843 (2.887,11.828)	7.516 (3.557,15.882)	0.000	2.992 (1.753,5.106)
Multivariable Model 1	1.705 (0.911,3.192)	2.223 (1.170,4.226)	5.413 (2.621,11.176)	7.261 (3.375,15.625)	0.000	2.849 (1.677,4.840)
Multivariable Model 2	1.516 (0.785,2.928)	1.939 (0.989,3.803)	4.629 (2.179,9.832)	7.072 (3.191,15.673)	0.000	1.008 (1.004,1.012)
16:0 (palmitic acid)						
Univariable Model	0.953 (0.519,1.749)	1.623 (0.868,3.034)	2.826 (1.449,5.510)	4.071 (1.987,8.343)	0.000	1.547 (1.094,2.186)
Multivariable Model 1	0.997 (0.531,1.870)	1.593 (0.828,3.066)	2.618 (1.306,5.248)	4.076 (1.924,8.634)	0.000	1.503 (1.056,2.139)
Multivariable Model 2	0.995 (0.515,1.920)	1.496 (0.755,2.965)	2.312 (1.111,4.811)	3.889 (1.783,8.481)	0.002	1.000 (1.000,1.000)
18:0 (stearic acid)						
Univariable Model	0.706 (0.380,1.312)	1.218 (0.640,2.318)	1.473 (0.765,2.837)	1.540 (0.795,2.983)	0.104	0.963 (0.794,1.169)
Multivariable Model 1	0.680 (0.359,1.285)	1.040 (0.528,2.048)	1.366 (0.694,2.688)	1.375 (0.684,2.763)	0.218	0.941 (0.771,1.149)
Multivariable Model 2	0.676 (0.347,1.316)	1.121 (0.545,2.307)	1.264 (0.621,2.572)	1.638 (0.785,3.418)	0.170	1.000 (1.000,1.000)
VLSFAs						
20:0 (arachidic acid)						
Univariable Model	1.170 (0.620,2.206)	1.282 (0.675,2.436)	1.462 (0.765,2.797)	1.089 (0.579,2.050)	0.814	1.030 (0.827,1.282)
Multivariable Model 1	1.317 (0.681,2.547)	1.287 (0.664,2.493)	1.625 (0.825,3.204)	1.080 (0.559,2.084)	0.663	1.018 (0.810,1.279)
Multivariable Model 2	1.537 (0.763,3.093)	1.668 (0.820,3.394)	1.919 (0.936,3.936)	1.438 (0.716,2.889)	0.459	1.005 (0.992,1.018)
22:0 (behenic acid)						
Univariable Model	1.380 (0.725,2.629)	1.033 (0.550,1.940)	1.327 (0.700,2.516)	1.260 (0.663,2.395)	0.803	1.732 (0.853,3.518)
Multivariable Model 1	1.334 (0.680,2.618)	0.934 (0.487,1.791)	1.260 (0.652,2.435)	1.159 (0.595,2.256)	0.813	1.877 (0.903,3.905)
Multivariable Model 2	1.578 (0.773,3.220)	1.024 (0.516,2.031)	1.354 (0.678,2.704)	1.448 (0.718,2.918)	0.617	1.072 (1.002,1.147)
24:0 (lignoceric acid)						
Univariable Model	0.898 (0.473,1.707)	1.099 (0.571,2.116)	0.825 (0.437,1.559)	1.020 (0.531,1.960)	0.917	1.182 (0.876,1.596)
Multivariable Model 1	0.853 (0.440,1.654)	1.112 (0.564,2.189)	0.813 (0.420,1.572)	1.127 (0.569,2.234)	0.815	1.250 (0.895,1.747)
Multivariable Model 2	0.954 (0.473,1.922)	1.155 (0.567,2.354)	0.816 (0.404,1.646)	1.370 (0.661,2.841)	0.665	1.061 (0.986,1.141)

LSFAs, long-chain saturated fatty acids; VLSFAs, Very-long-chain saturated fatty acids; Ref, reference.

Quintile1 is used as reference.

Multivariable Model 1 was adjusted for age, gender, duration of diabetes, alcohol intake.

Multivariable Model 2 was adjusted for age, gender, duration of diabetes, alcohol intake, body mass index, waist circumference.

### Additive interactions between LSFAs and IR for NAFLD

Additive interaction was further tested between myristic acid (14:0)>72.83 μmol/L and IR>4.89 for NAFLD risk. We found that co-presence of both myristic acid (14:0)>72.83 μmol/L and IR>4.89 greatly increased the risk when myristic acid (14:0)>72.83μmol/L alone (2.915, 1.493-5.691) or IR>4.89 alone (1.480, 0.848-2.582) for NAFLD to 9.691 (4.113-22.833). The additive interaction measures were significantly different (RERI: 6.296, -1.571-14.164); AP: 0.650, 0.329-0.971); S: 3.629, 1.166-11.298)).

In addition, co-presence of both palmitic acid (16:0)>3745.43μmol/L and IR>4.89 greatly increased risk when palmitic acid (16:0)>3745.43μmol/L alone (1.883, 0.991-3.576) or IR>4.89 alone (1.467, 0.840,2.561) for NAFLD to 6.518(2.860-14.854). The additive interaction measures were significantly different (RERI: 4.169, -0.835,9.174; AP: 0.640, 0.324,0.956); S: 4.090, 1.133,14.764) (**Table 3**).

**Table 3:** Odds ratio of saturated fatty acids and additive interaction with insulin resistance for nonalcoholic fatty liver disease.

	Unadjusted ^#^		Adjusted ^##^	
	OR (95%CI)	*p*-value	OR (95%CI)	*p*-value
Additive interaction models			
14:0 (myristic acid) μmol/L and IR			
14:0≤72.83 & IR≤4.89	Ref		Ref	
14:0≤72.83 & IR>4.89	1.467 (0.887,2.426)	0.136	1.480 (0.848,2.582)	0.168
14:0>72.83 & IR≤4.89	2.680 (1.439,4.989)	0.002	2.915 (1.493,5.691)	0.002
14:0>72.83 & IR>4.89	10.136 (4.560,22.528)	0.000	9.691 (4.113,22.833)	0.000
RERI	6.989 (-0.772,14.751)		6.296 (-1.571,14.164)	
AP	0.690 (0.418,0.961)		0.650 (0.329,0.971)	
S	4.256 (1.414,12.810)		3.629 (1.166,11.298)	
16:0 (palmitic acid) μmol/L and IR			
16:0≤3745.43 & IR≤4.89	Ref		Ref	
16:0≤3745.43 & IR>4.89	1.386 (0.836,2.298)	0.205	1.467 (0.840,2.561)	0.178
16:0>3745.43 & IR≤4.89	1.776 (0.982,3.211)	0.058	1.883 (0.991,3.576)	0.053
16:0>3745.43 & IR>4.89	7.706 (3.578,16.598)	0.000	6.518 (2.860,14.854)	0.000
RERI	5.544 (-0.068,11.156)		4.169 (-0.835,9.174)	
AP	0.719 (0.482,0.957)		0.640 (0.324,0.956)	
S	5.771 (1.592,20.926)		4.090 (1.133,14.764)	

OR, odds ratio; CI, confidence interval;14:0, myristic acid; 16:0, Palmitic acid; IR, insulin resistance; Ref, reference.

^#^Not adjusted for any other variables.

^##^Adjusted for age, gender, duration of diabetes, alcohol intake, body mass index, waist circumference.

Significant elative excess risk due to interaction (RERI) >0, attributable proportion due to interaction (AP) >0 or synergy index (S) >1 indicates a significant additive interaction.

### Sensitivity analysis

After exclusion of participants with a history of alcohol use (n=45), the effect sizes of myristic acid (14:0) and palmitic acid (16:0), and their ratios for NAFLD remained stable and significant in univariable and multivariable analyses (**Table 4**).

**Table 4:** Odds ratios (and 95% CIs) for nonalcoholic fatty liver disease by quintile of saturated fatty acids after exclusion of patients with a history of alcohol use.

Variables	Quintile 2	Quintile 3	Quintile 4	Quintile 5	*p* for trend	Per 1 SD
LSFAs						
14:0 (myristic acid)						
Univariable Model	1.869 (0.960,3.638)	2.861 (1.450,5.647)	5.677 (2.733,11.792)	8.516 (3.774,19.217)	0.000	3.816 (2.139,6.806)
Multivariable Model 1	1.811 (0.918,3.573)	2.588 (1.293,5.181)	5.387 (2.557,11.353)	8.481 (3.702,19.428)	0.000	3.762 (2.112,6.701)
Multivariable Model 2	1.603 (0.776,3.312)	2.236 (1.070,4.673)	4.560 (2.082,9.987)	8.687 (3.634,20.768)	0.000	3.597 (2.030,6.375)
16:0 (palmitic acid)						
Univariable Model	0.901 (0.468,1.735)	1.335 (0.692,2.577)	2.836 (1.365,5.891)	3.845 (1.780,8.307)	0.000	1.489 (1.022,2.170)
Multivariable Model 1	0.901 (0.459,1.769)	1.340 (0.678,2.651)	2.482 (1.171,5.264)	3.876 (1.746,8.602)	0.001	1.419 (0.968,2.080)
Multivariable Model 2	0.866 (0.424,1.768)	1.208 (0.586,2.489)	2.156 (0.968,4.799)	3.679 (1.595,8.484)	0.004	1.364 (0.960,1.940)
18:0 (stearic acid)						
Univariable Model	0.624 (0.322,1.208)	1.167 (0.580,2.348)	1.445 (0.713,2.930)	1.471 (0.726,2.979)	0.082	0.945 (0.769,1.161)
Multivariable Model 1	0.602 (0.306,1.183)	0.949 (0.459,1.961)	1.314 (0.638,2.707)	1.322 (0.633,2.760)	0.161	0.923 (0.743,1.146)
Multivariable Model 2	0.564 (0.276,1.154)	0.999 (0.456,2.190)	1.282 (0.594,2.766)	1.617 (0.738,3.540)	0.078	0.995 (0.805,1.230)
VLSFAs						
20:0 (arachidic acid)						
Univariable Model	1.103 (0.563,2.160)	1.196 (0.609,2.350)	1.619 (0.790,3.317)	0.939 (0.481,1.830)	0.633	0.987 (0.797,1.221)
Multivariable Model 1	1.259 (0.629,2.518)	1.197 (0.601,2.384)	1.758 (0.839,3.681)	0.914 (0.460,1.815)	0.492	0.967 (0.780,1.199)
Multivariable Model 2	1.482 (0.704,3.118)	1.530 (0.728,3.215)	2.162 (0.982,4.758)	1.308 (0.625,2.735)	0.423	1.067 (0.847,1.344)
22:0 (behenic acid)						
Univariable Model	1.808 (0.895,3.652)	1.184 (0.609,2.300)	1.312 (0.669,2.573)	1.372 (0.696,2.705)	0.572	1.532 (0.710,3.307)
Multivariable Model 1	1.568 (0.763,3.221)	1.026 (0.518,2.032)	1.166 (0.586,2.322)	1.233 (0.613,2.478)	0.762	1.590 (0.723,3.495)
Multivariable Model 2	1.871 (0.866,4.042)	1.156 (0.558,2.394)	1.300 (0.623,2.713)	1.642 (0.779,3.462)	0.495	2.133 (0.890,5.113)
24:0 (lignoceric acid)						
Univariable Model	0.729 (0.371,1.432)	1.074 (0.521,2.211)	0.686 (0.348,1.353)	1.020 (0.499,2.086)	0.583	1.108 (0.828,1.482)
Multivariable Model 1	0.711 (0.356,1.421)	1.066 (0.510,2.229)	0.646 (0.322,1.298)	1.113 (0.533,2.326)	0.430	1.125 (0.810,1.563)
Multivariable Model 2	0.805 (0.383,1.694)	1.113 (0.508,2.438)	0.632 (0.298,1.342)	1.426 (0.643,3.164)	0.282	1.263 (0.865,1.845)

LSFAs, long-chain saturated fatty acids; VLSFAs, Very-long-chain saturated fatty acids; Ref, reference.

Quintile1 is used as reference.

Multivariable Model 1 was adjusted for age, gender, duration of diabetes.

Multivariable Model 2 was adjusted for age, gender, duration of diabetes, body mass index, waist circumference.

## Discussion

A study has shown that the median age of NAFLD patients in China is younger than it in other countries, which means the prevalence of advanced liver disease in China is not too high now, however, with the progress of aging, a foreseeable trend is that the associated burden of disease will increase dramatically. In addition, obesity is an independent risk factor for the onset of diabetes, and a fact can’t be ignored is that the obesity rate in China is still increasing. Against the above background, the prevalence of diabetes in China will undoubtedly increase in the future. It is imperative to conduct research on the above-mentioned issues (41).

In our cross-sectional study, we found myristic acid (14:0) and palmitic acid (16:0) were associated with the risk of NAFLD in Chinese patients with T2D. In addition, there was a significant additive interaction between (high myristic acid (14:0) or palmitic acid (16:0)) and high IR. High IR amplifies the degree of effect of myristic acid (14:0) and palmitic acid (16:0) on NAFLD. A cross-sectional study of 320 participants including 240 NAFLD and 80 healthy individuals showed that myristic acid (14:0) was positively associated with NAFLD (42); A small study in 59 participants reported that myristic acid (14:0) and palmitic acid (16:0) powerfully raise serum total cholesterol and low-density lipoprotein cholesterol (LDL-C) levels in a human trial involving dietary SFAs (43). Martínez showed that the consumption of diets enriched in both myristic acid (14:0) and palmitic acid (16:0) could cause NASH related to lipodystrophy (44). Consistent with these findings, we found that both myristic acid (14:0) and palmitic acid (16:0) were associated with the risk of NAFLD in Chinese T2D patients. Myristic acid (14:0), a not lipotoxic SFA, is highly abundant in copra and palmist oils. Palmitic acid (16:0), a lipotoxic SFA, targets different organelles (including ER and mitochondria) and mediates hepatocyte apoptosis (45, 46). Palmitic acid (16:0) and myristic acid (14:0) can stimulate ceramide synthesis (44, 47). Research shows that that ceramide is one of the most active lipid second messengers that inhibit the insulin signaling pathway (48, 49). Ceramides can promote liver IR and inhibit Akt signaling pathway, and IR increases lipolysis and promotes the delivery of free fatty acids to the liver (50),which is an example of a vicious circle.

The present study has potential clinical and mechanistic implications. There are many people with T2D in China who are at higher risk for NAFLD (51). A growing number of people are expected to have NAFLD in the future. So, it is extremely important to accurately diagnose and treat cases at individual levels. Our study generates new ideas for the diagnosis of NAFLD in T2D patients. Moreover, our findings provide some new hypotheses for scientists to explore the molecular mechanism of liver fat accumulation in diabetes. The study suggests that myristic acid (14:0) and palmitic acid (16:0), especially combined with high IR, might have a profound impact on the diagnosis and treatment of NAFLD with T2D in Chinese individuals if these findings can be replicated in cohort studies, especially, in China.

The present study also had several limitations. First, the main limitation of the study is the cross-sectional design, no definite causal association between LSFAs and NAFLD can be derived. Therefore, the present study shows an urgent need to validate these findings in other cohort studies. Second, our participants were in-patients with T2D and they may have more severe T2D and are at greater risk of NAFLD than those non-hospitalized patients with T2D. Although we made the careful adjustment for disease severity, we still need further studies to validate these findings in carefully designed cohorts. Third, physical activity and diet in participants with NAFLD might be different from subjects without NAFLD. We did not collect these data in this survey. Nevertheless, there were associations between physical activity and diet and BMI,WC and even the content of the fatty acids studied. Therefore, careful adjustment for WC and BMI may have partially eliminated the confounding effects of diet and physical activity. Fourth, the study population was collected from the Dalian of China alongside a small sample size. Therefore, our conclusions might lack generality to the whole population. A large sample of longitudinal studies is necessary to validate our conclusions. Thus, the reported effect sizes of the OR and the additive interaction between high myristic acid (14:0) and palmitic acid (16:0) and high IR might underestimate their true effect sizes. Finally, genetic factors are one of the factors that contribute to the development of diabetes, which can also lead to differences in metabolic levels between different patients. However, we have not been able to go down to the genetic level to do stratification studies is also one of the limitations.

## Conclusion

In conclusion, our findings showed that myristic acid (14:0) and palmitic acid (16:0) were positively associated with the risk of NAFLD in Chinese T2D patients. High IR amplified the effect of high myristic acid (14:0) and high palmitic acid (16:0) on NAFLD. As our findings came from a cross-sectional study, further cohort studies are warranted to confirm our findings in Chinese T2D patients and other populations.

## Data availability statement

The raw data supporting the conclusions of this article will be made available by the authors, without undue reservation.

## Ethics statement

The studies involving human participants were reviewed and approved by The First Affiliated Hospital of Jinzhou Medical University. Written informed consent for participation was not required for this study in accordance with the national legislation and the institutional requirements.

## Author contributions

The above two authors completed the writing of this article, and they unanimously decided to submit the manuscript to your journal. L-PJ completed the writing of the article, including data collation, analysis, article writing and revision. H-ZS conceived and designed the article and made suggestions on the first draft until satisfied. All authors contributed to the article and approved the submitted version.
